# Electrodiffusion-Mediated Swelling of a Two-Phase Gel Model of Gastric Mucus

**DOI:** 10.3390/gels4030076

**Published:** 2018-09-06

**Authors:** Owen L. Lewis, James P. Keener, Aaron L. Fogelson

**Affiliations:** 1 Department of Mathematics, Florida State University, Tallahassee, FL 32306-4510, USA; olewis@math.utah.edu; 2 Departments of Mathematics and Bioengineering, University of Utah, Salt Lake City, UT 84112, USA; keener@math.utah.edu

**Keywords:** polyelectrolyte gel, mathematical model, gel swelling

## Abstract

Gastric mucus gel is known to exhibit dramatic and unique swelling behaviors in response to the ionic composition of the hydrating solution. This swelling behavior is important in the maintenance of the mucus layer lining the stomach wall, as the layer is constantly digested by enzymes in the lumen, and must be replenished by new mucus that swells as it is secreted from the gastric wall. One hypothesis suggests that the condensed state of mucus at secretion is maintained by transient bonds with calcium that form crosslinks. These crosslinks are lost as monovalent cations from the environment displace divalent crosslinkers, leading to a dramatic change in the energy of the gel and inducing the swelling behavior. Previous modeling work has characterized the equilibrium behavior of polyelectrolyte gels that respond to calcium crosslinking. Here, we present an investigation of the dynamic swelling behavior of a polyelectrolytic gel model of mucus. In particular, we quantified the rate at which a globule of initially crosslinked gel swells when exposed to an ionic bath. The dependence of this swelling rate on several parameters was characterized. We observed that swelling rate has a non-monotone dependence on the molarity of the bath solution, with moderate concentrations of available sodium inducing the fastest swelling.

## 1. Introduction

Gastric mucus is a polyelectrolyte gel which serves an important biological function. It is generally accepted that the gastric mucus layer provides a protective barrier between the stomach wall and the stomach interior, shielding the wall from acid and digestive enzymes and preventing auto-digestion of the stomach epithelium. However, there are several outstanding questions regarding the maintenance of the gastric barrier. Digestive enzymes within the stomach are constantly degrading the mucus layer from the inside-out, cleaving the mucin glycoproteins and destroying the entangled network which makes up the mucus gel [1]. This degradation of the network must be balanced by secretion of fresh mucus from the stomach wall to maintain a stable layer.

Fresh mucus is secreted from epithelial cells on the stomach wall via exocytosis of microscopic vesicles filled with densely packed mucin polymers. This dense packing of negatively charged polymers is shielded by an abundance of divalent calcium ions within the vesicle [2]. When the contents of these vesicles are exposed to an aqueous environment, the mucus swells dramatically [3]. However, this swelling behavior is known to depend on environmental pH and ionic composition [4]. In particular, when dense mucus is secreted into a deionized environment, the dramatic swelling event does not occur [3]. Experiments have shown that swelling is accompanied by a massive (and rapid) transport of monovalent cations (such as sodium or hydrogen) into the densely packed mucus in exchange for the divalent calcium [5], removing the efficient charge shielding. It is possible that the divalent nature of calcium ions provides an explanation for the observed behavior. Due to having a charge of 2+, calcium can form two bonds with the negatively charged mucin polymers, effectively forming a “crosslink”. Even though these crosslinks are much less stable than (for example) one potentially formed by covalent bonds [6,7,8,9,10], large numbers of crosslinks may allow for very dense network configurations. Conversely, replacing calcium with monovalent sodium removes the ability to form crosslinks, potentially causing rapid hydration of the gel.

The dynamics of swelling hydrogels have been studied for over seventy years, using theoretical frameworks of increasing sophistication [11]. However, the proposed mechanism must necessarily depend on electrodiffusive transport of ionic species, motion of the glycoprotein network and hydrating fluid, and chemical interactions between the network and dissolved ions. All of these processes are coupled and affect one another. In [12], the authors derived from first principles a model describing the transport of mono- and divalent ionic species which bind and unbind to a gel-like material described by a two-phase mixture model. The equilibrium behavior of the model is explored in [12], and the linear behavior near equilibria is explored in [13]. We present here the first general investigation of the dynamic swelling behavior of this model. In particular, we attempted to quantify the rate of swelling of a polyelectrolyte gel material as a function of the parameters which govern its chemical interaction with dissolved ions.

## 2. Mathematical Model

In this work, we choose to model a mucus gel using a two-phase framework. Similar frameworks have been used in a wide variety of biological contexts, including the modeling of blood clots, cellular mechanics, and bacterial biofilms [14,15,16,17]. In our framework, the local composition of the complex gel material is described by the volume fractions of two distinct phases which we call network (denoted with subscript *n*) and solvent (subscript *s*). The network represents the entangled mesh of polymeric proteins which give rise to the gel, while the solvent represents the interstitial hydrating fluid. Each material is allowed to move with its own distinct velocity. Dissolved ionic species are allowed to move through the solvent phase, and bind/unbind to/from the network phase.

### 2.1. Gel Evolution

As is standard in a two-phase model, there are four equations for the composite material composed of network and solvent. The first two equations describe conservation of mass of the solvent and network phases, respectively:

(1) $$\frac{\partial }{\partial t}\theta_{n}+\nabla \cdot \left(\theta_{n}{\vec{u}}_{n}\right)=0,$$

(2) $$\frac{\partial }{\partial t}\theta_{s}+\nabla \cdot \left(\theta_{s}{\vec{u}}_{s}\right)=0.$$

Here, $\theta_{n}$ and $\theta_{s}$ are the volume fractions of network and solvent, respectively, and ${\vec{u}}_{n}$ and ${\vec{u}}_{s}$ are their respective velocities. By definition, the volume fractions satisfy the constraint $\theta_{s}\left(\vec{x},t\right)+\theta_{n}\left(\vec{x},t\right)=1$. As a consequence of this constraint, Equations (1) and (2) imply that the volume-averaged incompressibility condition

(3) $$\nabla \cdot \left(\theta_{n}{\vec{u}}_{n}+\theta_{s}{\vec{u}}_{s}\right)=0,$$

must hold. The velocity fields of each phase are determined by conservation of momentum. Because the spatial scale of interest is small (microns) and the velocities of the solvent and network are low, it is reasonable to ignore inertial forces. Hence, momentum balance reduces to force balance equations for each phase,

(4) $$\nabla \cdot \left(\theta_{n}{\underline{\sigma }}_{n}\right)-\xi \theta_{n}\theta_{s}\left({\vec{u}}_{n}-{\vec{u}}_{s}\right)-\theta_{n}\nabla \mu_{n}-\theta_{n}\nabla p=0,$$

(5) $$\nabla \cdot \left(\theta_{s}{\underline{\sigma }}_{s}\right)-\xi \theta_{n}\theta_{s}\left({\vec{u}}_{s}-{\vec{u}}_{n}\right)-\theta_{s}\nabla \mu_{s}-\theta_{n}\nabla p-\theta_{s}\left(\sum_{j}\phi_{j}\nabla \mu_{j}\right)=0.$$

Here, ${\underline{\sigma }}_{n}$ and ${\underline{\sigma }}_{s}$ are stress tensors which encapsulate the internal stresses within the network and solvent, respectively; ξ is the coefficient of the drag force that arises whenever there is relative motion between the two materials; $\mu_{s}$, $\mu_{n}$, and $\mu_{j}$ for j≥3 are chemical potentials that act on the solvent, network, and ionic species, respectively; and *p* is the hydrodynamic pressure. The final term in Equation (5) replaces the drag forces between the ions and the solvent. In effect, the forces from the ionic potentials are transferred to the solvent because the only forces that act on the ions of type *j* are that from $\nabla \mu_{j}$ and the drag between the solvent and these ions, and these forces must sum to zero. The pressure *p* is determined by the incompressibility constraint in Equation (3). In Equation (5), $\phi_{j}$ is the ratio of the number density of ion *j* to the number density of water particles. This ionic contribution is valid in the limit that ions are dilute in the solvent.

The internal stresses within each phase must be given by a constitutive law. In this work, we assume that both the solvent and network exhibit a viscous response (though the modeling framework is adaptable to account for elastic or viscoelastic constitutive laws as well). In one spatial dimension, the stress tensor for each phase reduces to

(6) $${\underline{\sigma }}_{i}=\nu_{i}\frac{\partial {\vec{u}}_{i}}{\partial x},\;i=s,n,$$

where $\nu_{i}$ is the viscosity of the respective phase.

### 2.2. Dissolved Ion Evolution

The ionic species dissolved within the solvent phase are each subject to a Nernst–Planck type equation, modified to account for diffusion through a fluid of non-uniform volume fraction. Cations may bind to the network via mass-action type reactions, while anions may not. We are specifically interested in the behavior of the model when we have three distinct types of ions present: a monovalent cation (which we call sodium), a divalent cation (calcium), and a monovalent anion (chloride).

(7) $$\begin{array}{cc} \frac{\partial }{\partial t}c_{\mathrm{Na}}+\nabla \cdot \left(c_{\mathrm{Na}}{\vec{u}}_{s}\right) & =\frac{1}{\theta_{s}}\nabla \cdot \left(D_{\mathrm{Na}}\theta_{s}\left(\nabla c_{\mathrm{Na}}+z_{\mathrm{Na}}c_{\mathrm{Na}}\nabla \Psi \right)\right)-k_{\mathrm{Na}}^{\mathrm{on}}\theta_{n}Mc_{\mathrm{Na}}+k_{\mathrm{Na}}^{\mathrm{off}}\theta_{s}^{2}b_{\mathrm{Na}}, \end{array}$$

(8) $$\begin{array}{cc} \frac{\partial }{\partial t}c_{\mathrm{Ca}}+\nabla \cdot \left(c_{\mathrm{Ca}}{\vec{u}}_{s}\right) & =\frac{1}{\theta_{s}}\nabla \cdot \;\left(D_{\mathrm{Ca}}\theta_{s}\left(\nabla c_{\mathrm{Ca}}+z_{\mathrm{Ca}}c_{\mathrm{Ca}}\nabla \Psi \right)\right)-k_{\mathrm{Ca}}^{\mathrm{on}}\theta_{n}Mc_{\mathrm{Ca}}+k_{\mathrm{Ca}}^{\mathrm{off}}\theta_{s}^{2}b_{\mathrm{Ca}}, \end{array}$$

(9) $$\begin{array}{cc} \frac{\partial }{\partial t}c_{\mathrm{Cl}}+\nabla \cdot \left(c_{\mathrm{Cl}}{\vec{u}}_{s}\right) & =\frac{1}{\theta_{s}}\nabla \cdot \left(D_{\mathrm{Cl}}\theta_{s}\left(\nabla c_{\mathrm{Cl}}+z_{\mathrm{Cl}}c_{\mathrm{Cl}}\nabla \Psi \right)\right). \end{array}$$

Here, $c_{j}$ is the concentration of species *j* measured in units of moles per liter *solvent* volume, $D_{j}$ is that ion’s diffusion coefficient, $z_{j}$ its valence, and Ψ is the electric potential measured in terms of thermal voltage RT/F (*R*: ideal gas constant; *T*: absolute temperature; and *F*: Faraday constant). The parameter $k_{j}^{\mathrm{on}}$ is the binding rate of ion *j* to the gel network, while $k_{j}^{\mathrm{off}}$ is the corresponding unbinding rate. The ratio $K_{j}=k_{j}^{\mathrm{off}}/k_{j}^{\mathrm{on}}$ is the dissociation constant of that cation.

### 2.3. Bound Ion Evolution

Cations that are bound to the mucus network advect with the network velocity. The concentration of bound ions may also change through binding and unbinding reactions described by

(10) $$\begin{array}{cc} \frac{\partial }{\partial t}b_{\mathrm{Na}}+\nabla \cdot \left(b_{\mathrm{Na}}{\vec{u}}_{n}\right) & =k_{\mathrm{Na}}^{\mathrm{on}}\theta_{s}Mc_{\mathrm{Na}}-k_{\mathrm{Na}}^{\mathrm{off}}\theta_{s}^{2}b_{\mathrm{Na}}, \end{array}$$

(11) $$\begin{array}{cc} \frac{\partial }{\partial t}b_{\mathrm{Ca}}+\nabla \cdot \left(b_{\mathrm{Ca}}{\vec{u}}_{n}\right) & =k_{\mathrm{Ca}}^{\mathrm{on}}\theta_{s}Mc_{\mathrm{Ca}}-k_{\mathrm{Ca}}^{\mathrm{off}}\theta_{s}^{2}b_{\mathrm{Ca}}-\frac{1}{2}k_{\mathrm{Ca}}^{\mathrm{on}}Mb_{\mathrm{Ca}}+2k_{\mathrm{Ca}}^{\mathrm{off}}b_{C2}, \end{array}$$

(12) $$\begin{array}{cc} \frac{\partial }{\partial t}b_{C2}+\nabla \cdot \left(b_{C2}{\vec{u}}_{n}\right) & =\frac{1}{2}k_{\mathrm{Ca}}^{\mathrm{on}}Mb_{\mathrm{Ca}}-2k_{\mathrm{Ca}}^{\mathrm{off}}b_{C2}. \end{array}$$

Here, $b_{\mathrm{Na}}$ is the concentration of sodium bound to the network, measured in moles per *total* volume. Because calcium is divalent, it may bind twice, and therefore exists in one of two bound states: singly bound ($b_{\mathrm{Ca}}$) and doubly bound ($b_{C2}$). The quantity *M* is the concentration of negative charge on the mucus network which is *not* bound to a cation; it is determined by the relationship

(13) $$\tilde{z}\theta_{n}=M+b_{\mathrm{Na}}+b_{\mathrm{Ca}}+2b_{C2},$$

where $\tilde{z}$ is the density (per network volume) of negative sites on a unit of network. We are assuming that negative charge on the network and binding sites which cations may occupy exist in a one-to-one ratio.

### 2.4. Driving Potentials

The potentials which act on the volume-occupying species (network and solvent) account for entropic, long-range electrostatic, and short-range internal energy effects. The potentials acting directly on the solvent are given by

(14) $$\frac{\mu_{s}}{k_{B}T}=\underset{I}{\underset{︸}{\ln\left(\theta_{s}\right)+\left(1-\frac{1}{N}\right)\theta_{n}-\sigma_{I}}}+\underset{II}{\underset{︸}{\left(\frac{I}{2}\theta_{n}^{2}+\mu_{s}^{0}\right)}},$$

and the potentials acting directly on the network are given by

(15) $$\frac{\mu_{n}}{k_{B}T}=\underset{I}{\underset{︸}{\frac{1}{N}\ln\left(\theta_{n}\right)+\left(\frac{1}{N}-1\right)\theta_{s}}}+\underset{II}{\underset{︸}{\left(\frac{I}{2}\theta_{s}^{2}+\mu_{n}^{0}\right)}}-\underset{III}{\underset{︸}{\tilde{z}\Psi }}.$$

Terms in Equations (14) and (15) labeled (I) represent entropic effects, and capture osmotic pressure acting on the solvent. Terms labeled (II) represent potentials due to short range interactions and generalize the standard Flory–Huggins mixture theory. We discuss the factor I in more detail below. Finally, the term labeled (III) represents the potential due to the charged nature of the mucus network. Here, $k_{B}$ is the Boltzmann constant and *N* is the number of monomers in a typical polymer chain of mucin. The term $\sigma_{I}$ is the total ionic molality, given by

(16) $$\sigma_{I}=\sum_{j}\phi_{j},$$

where, again, $\phi_{j}=c_{j}/c_{\mathrm{water}}$ is the concentration of ion *j* divided by the standard molarity of water ($c_{\mathrm{water}}=55.5$ M).

Because the ionic species are regarded as massless, the forces on each species are in balance. Thus, the chemical potential force and the solvent drag force on each ionic species sum to zero. Since the drag force the ions exert on the solvent is the opposite of that the solvent exerts on the ions, the net effect is that the chemical potential forces appear to act on the solvent itself (as incorporated in the last term of Equation (5)). The potentials which act on dissolved ions are given by

(17) $$\frac{\mu_{j}}{k_{B}T}=\underset{I}{\underset{︸}{\ln\left(\phi_{j}\right)+1-2\sigma_{I}}}+\underset{II}{\underset{︸}{z_{j}\Psi }},$$

where $z_{j}$ is the valence of ion *j*. Equation (17) accounts for both entropic (I) and electric (II) potentials.

We now return to the term I, which appears in Term (II) in Equations (14) and (15). We refer to this as the “interaction parameter”, and note that it is somewhat analogous to a typical Flory interaction parameter [11]. However, here I is not a constant; it is given by

(18) $$I=6\left(\epsilon_{1}+\epsilon_{2}\right)-2\left(1-\frac{1}{N}\right)\epsilon_{1}-\epsilon_{1}\alpha ,$$

where $\epsilon_{1}$ and $\epsilon_{2}$ are constants described below. The quantity $\alpha (\vec{x},t)$ is the ratio of the concentration of current crosslinks within the network ($b_{C2}\left(\vec{x},t\right)$) to the maximum concentration of crosslinks possible ($\tilde{z}\theta_{n}\left(\vec{x},t\right))/2$):

(19) $$\alpha =\frac{2b_{C2}}{\tilde{z}\theta_{n}}.$$

Because α may vary spatially and temporally as the concentration of doubly bound calcium and network volume fraction do, so may I. Thus, our modeling framework is capable of describing a gel network with a “Flory interaction parameter” that varies spatiotemporally in response to the local ionic concentrations.

Finally, terms $\mu_{n}^{0}$ and $\mu_{s}^{0}$ (in Equations (14) and (15)) are the so-called “standard free energy” of the mucus phase and solvent phase, respectively. The solvent term $\mu_{s}^{0}$ is a constant, and therefore does not affect the behavior of the system (as only gradients of potentials appear in the force balance). The network standard free energy is given by

(20) $$\mu_{n}^{0}=-3\epsilon_{3}+\epsilon_{4}\left(1-\frac{1}{N}\right)+\frac{\alpha }{2}\epsilon_{3}.$$

Note that the crosslinking fraction α appears in this expression. The parameters $\epsilon_{1}$, $\epsilon_{2}$, $\epsilon_{3}$, and $\epsilon_{4}$ are referred to as the interaction energies, and arise in a standard mean-field calculation of the internal energy associated with a given mucus/solvent mixture. A detailed derivation of this modeling framework, especially the potentials given in Equations (14), (15) and (17), may be found in [12].

## 3. Results

### 3.1. Numerical Experiments and Initial Conditions

**Figure 1:**
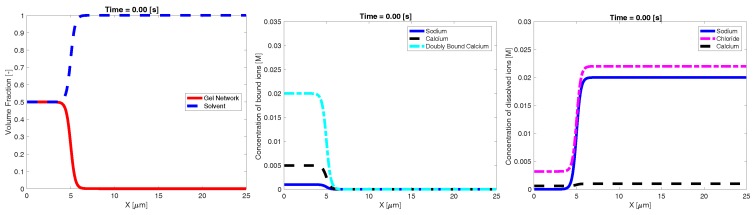
Example initial profiles of: volume fraction (**left**); bound ionic concentrations (**middle**); and dissolved ionic concentrations (**right**).

Our goal was to investigate the effect of the ambient ionic concentration on the dynamic swelling of high density network. To this end, we constructed initial profiles of volume fraction and ionic concentrations which represent a dense, highly crosslinked amount of network in a small region immersed in a fluid of known ionic composition. We refer to the region with appreciable volume fractions of network as the “inner” region, and initially placed it at the left end of the one-dimensional domain of width L=25 µm. The rest of the domain is referred to as the “bath” and initially has essentially no network and a prescribed ionic composition. To construct the initial profiles, we prescribed $\theta_{n}$, $\tilde{z}$ and the *total* amount of calcium, sodium, and chloride. Under the assumption that all species are spatially uniform, Equations (7) to (12) may be solved to determine the individual concentrations ($c_{j}$ and $b_{j}$) at which the binding and unbinding chemistry is at local equilibrium. This process was carried out separately for the inner region and the bath. We then used a hyperbolic tangent function (centered at x=5 µm) to construct spatial profiles that transition smoothly but sharply between the inner and bath concentrations. These profiles were used as the initial conditions for Equations (7) to (12). An example of the initial conditions can be seen in Figure 1. We simulated Equations (7) to (12) using no-flux boundary conditions for all species and zero Dirichlet conditions for both velocities (to represent a closed container) to investigate the dynamic swelling behavior of the network. Please see Appendix A.1 for details of the numerical techniques used.

In all simulations, the initial volume fraction of network, the mechanical and energetic properties of the network (the parameters $\mu_{s}$, $\mu_{n}$, ξ, $\epsilon_{1}$, $\epsilon_{2}$, $\epsilon_{3}$, $\epsilon_{4}$ and *N*), and the diffusion coefficients ($D_{j}$) of the ions were identical. The values of these parameters are listed in Table 1. In particular, the $\epsilon_{i}$ were chosen to produce a network which has a very weak propensity to swell when fully cross-linked, but swells quickly when no calcium crosslinking is present. When fully crosslinked (α=1), the network exhibits an interaction parameter I=0, which does not drive swelling at all (this is analogous to a Flory interaction parameter of zero). However, when there is no crosslinking (α=0), the network exhibits an interaction parameter I=−45, which is analogous to a large, negative Flory interaction parameter, and leads to rapid swelling. The parameter $\epsilon_{3}$ was chosen to be small and negative, as large negative values of this parameter can lead to gels which tend to phase separate, which is not a behavior we wanted to investigate. The final interaction energy $\epsilon_{4}$ does not impact the system dynamics, as it is simply an additive constant that produces no potential gradient. In all simulations, the bath concentration of sodium was large, while the calcium concentration was small. The chloride concentration was chosen to maintain electroneutrality. This choice was made to allow monovalent sodium from the bath to displace calcium crosslinks within the network.

We performed four major sets of experiments to investigate how two basic aspects of the network chemistry affect swelling behavior. Specifically, we investigated the regime where there is an extremely high density of binding sites on the network (referred to as “dense binding”), as well as the regime where this is a more moderate density of binding sites (referred to as “sparse binding”). Estimates for the density of cation binding sites on *salivary* mucus have been reported to be less than one molar [18]. However, the experiments in [19] suggest that gastric mucus may bind hydrogen ions in concentrations above one molar. Therefore, we assumed that a “sparse” network exhibits 0.1 mole of binding sites per liter of pure mucus ($\tilde{z}=0.1$) while a “dense” network has 1 mole of binding sites per liter of pure mucus ($\tilde{z}=1$). We also investigated the case where a network–calcium bond is chemically preferred to a network–sodium bond ($K_{\mathrm{Ca}}<K_{\mathrm{Na}}$), as well as the case where network–sodium bonds are preferred ($K_{\mathrm{Ca}}>K_{\mathrm{Na}}$). We refer to these cases as “calcium preferred” and “sodium preferred”, respectively. We investigated both cases, as estimates for the dissociation constants for both sodium and calcium vary over orders of magnitude depending on the experimental setup and type of mucus used [18,20]. Furthermore, we know of no reliable estimates for the individual kinetic rate constants for either ion. The steady state analysis in [12] assumed that the timescale of chemical reactions was fast compared to other timescales in the problem. Following in this spirit, we chose $k_{\mathrm{Ca}}^{\mathrm{on}}$, $k_{\mathrm{Ca}}^{\mathrm{off}}$, $k_{\mathrm{Na}}^{\mathrm{on}}$, and $k_{\mathrm{Na}}^{\mathrm{off}}$ to be large and to produce dissociation constants which are roughly in line with literature values ($10^{-4}$ or $10^{-3}$). We note that the phrase “calcium preferred” only describes the relative size of the two dissociation constants, and does not necessarily imply that there is actually more calcium bound to the network. The actual concentration of bound ions of each type depends on the chemistry, as well as the total amounts of each ion available for binding. To construct a network which is initially in a highly crosslinked state, we chose inner sodium and calcium concentrations that, for each case, yield a binding/unbinding equilibrium where most of the network is crosslinked (α>0.8). Chloride concentrations were then specified to maintain electroneutrality. In each of these four regimes, we carried out swelling experiments for a variety of bath concentrations of sodium. Again, chloride concentrations were adjusted to maintain electroneutrality. The inner and bath concentrations, as well as the binding/unbinding rates and binding site density used in each of these four cases are listed in Table 2.

### 3.2. Gel Swelling Experiments

**Figure 2:**
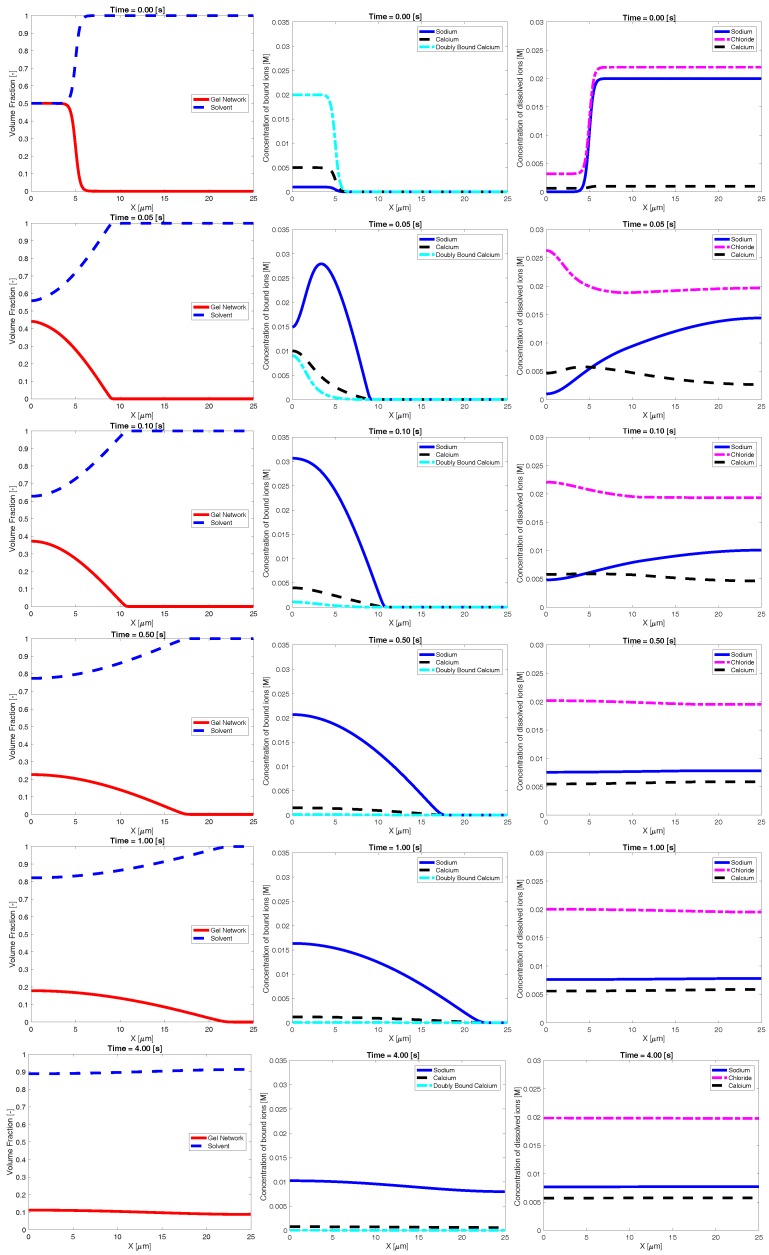
Example time evolution of: volume fraction (**first column**); bound ionic concentrations (**second column**); and dissolved ionic concentrations (**third column**).

Figure 2 shows an example time evolution of the network and solvent volume fraction, as well as dissolved and bound ion concentrations. The shown data were generated using a bath sodium concentration of 0.02 M with $K_{\mathrm{Ca}}>K_{\mathrm{Na}}$ and sparse binding sites. The initial condition was the same as that shown in Figure 1. The images show that, as time progresses, sodium from the bath diffuses into the inner region, reducing the bath concentration of sodium. As this occurs, the concentration of bound sodium drastically increases and propagates left, towards the interior region of dense network. This necessarily drives a decrease in the concentration of bound and doubly-bound calcium, and this release of bound calcium leads to an increase in the concentration of calcium dissolved in the solvent. As time progresses, the ionic concentrations (both bound and dissolved) approach uniform profiles. As the ions diffuse into and out of the region of network volume fraction, there is a rearrangement of the network phase. In particular, volume fraction of network flows from left (high density) to right. This decrease in maximum network density combined with an increase in the spatial region where network volume fraction exists is interpreted as swelling of the network phase. Eventually, over the course of several seconds, the network phase spreads across the domain and approaches a uniform steady state.

**Figure 3:**
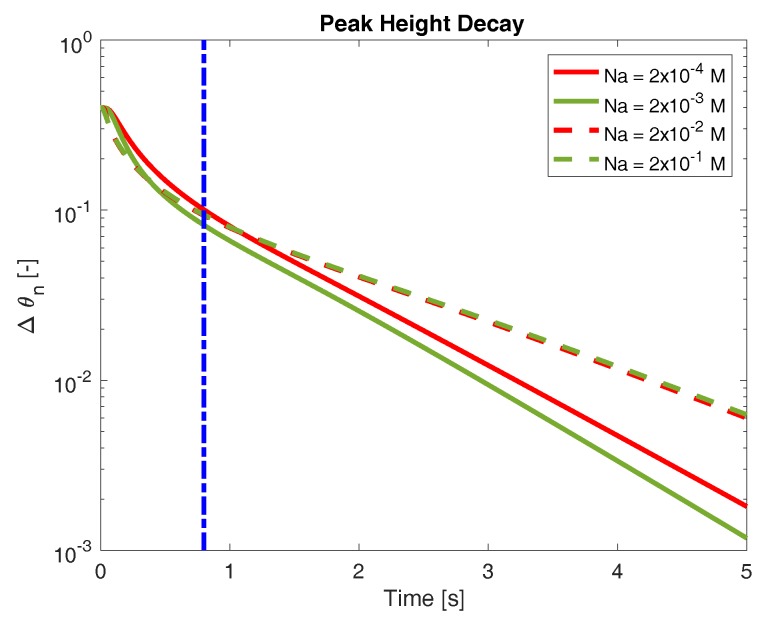
Decay of maximum network volume fraction to steady state for several concentrations of external sodium. Vertical dash-dot line indicates (roughly) the transition from early-time expansion behavior to long-time exponential behavior.

To quantitatively measure the rate at which the network phase swells, we track the maximum network volume fraction ($\theta_{n}^{\max}\left(t\right)$) as a function of time. Due to conservation of mass, the volume fraction of network at steady state (when the gel is completely uniform) may be determined directly from initial conditions. We denote this quantity as $\theta_{n}^{\mathrm{ss}}$ and note that, in these experiments, $\theta_{n}^{\mathrm{ss}}\approx 0.1$. We may then define the quantity

(21) $$\Delta \theta \left(t\right)=\theta_{n}^{\max}-\theta_{n}^{\mathrm{ss}}$$

as a measure of the network swelling behavior. As the network swells towards its final configuration, Δθ approaches zero. Figure 3 shows the time evolution of Δθ for several bath concentrations of sodium. All of these data were produced in the case when binding is sparse and sodium binding is preferred ($K_{\mathrm{Ca}}>K_{\mathrm{Na}}$). It is immediately apparent that the bath concentration of sodium influences the rate of swelling, although the nature of this influence is not necessarily simple. The rate at which Δθ decreases does not appear to be monotonic with respect to bath sodium concentrations (we return to this below). Furthermore, the effect of bath sodium on swelling rate varies depending on the time one considers. For example, at times prior to roughly 1 s, the network swells more quickly when the bath sodium concentration is 0.002 M (green solid curve in Figure 3) compared to when it is 0.02 M (red dashed curve). However, at longer time scales, this behavior reverses, and the network immersed in a 0.02 M sodium bath swells more rapidly. Finally, we note that, at longer times (greater than approximately 1 s), the behavior of Δθ appears exponential (linear on the semilog plot in Figure 3). Exponential swelling behavior has been predicted by numerous other studies. However, on time scales less than a second, the behavior of Δθ is distinct. This “short time” behavior is a novel prediction of the model, and something that we investigate further below. The transition between this “short time” behavior and the more obvious exponential decay is difficult to precisely quantify, but generically occurs sometime prior to 1 s. To focus on the short time swelling behavior, we turn to a separate quantification of swelling rate.

### 3.3. Front Propagation

For expositional purposes, suppose that a region of network of size $L^{*}\left(t\right)$ and *constant* volume fraction θ(t) swells to a new, steady state size *L* and volume fraction $\theta_{n}^{\mathrm{ss}}$. Conservation of mass stipulates that, at all points in time, $L^{*}\left(t\right)\theta \left(t\right)=L\theta_{n}^{\mathrm{ss}}$. If we then assume that volume fraction approaches its steady state value in an exponential manner

(22) $$\theta \left(t\right)-\theta_{n}^{\mathrm{ss}}\propto e^{-\gamma t},$$

then we may deduce that

(23) $$\Delta \left(L^{-1}\right)=\frac{1}{L^{*}\left(t\right)}-\frac{1}{L}\propto e^{-\gamma t}.$$

From Figure 2, it is clear that the network in our experiments does not have a spatially uniform profile, and thus does not meet the assumptions of the above calculation. However, this argument provides a scaling law for the size of the network globule which we investigate. Now, to define the width of the network globule in our experiments, we identify the location where network volume fraction is equal to 0.01

$$\theta_{n}\left(L^{*}\left(t\right)\right)=0.01.$$

**Figure 4:**
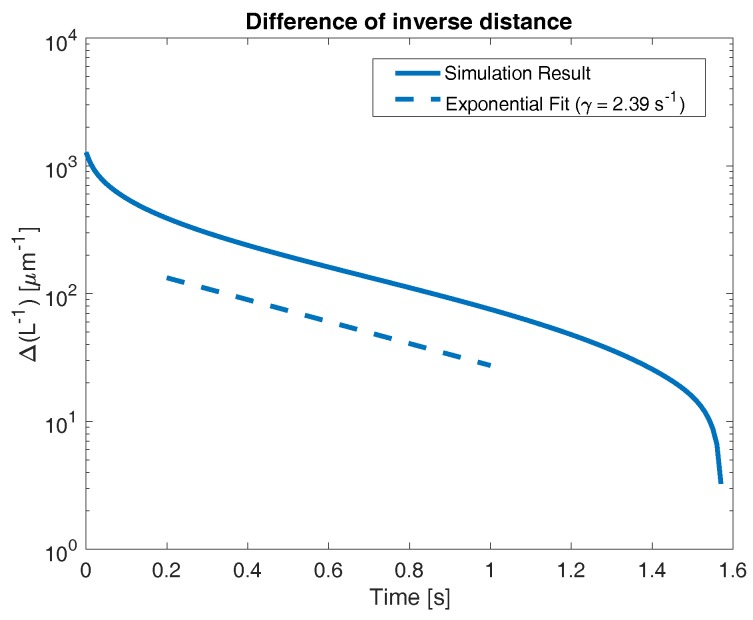
Time evolution of network front towards right wall (at x=L). Dashed line indicates exponential fit from t=0.2 to t=1 s. The slope of this line (γ=2.39 s${}^{-1}$) represents the early-time expansion rate of the network.

We refer to the location $L^{*}$ as the “front” of the network, as there is 99% solvent (by volume) in the space to the right of this point. We know that the steady state width of the network is L=25 µm, as it fills the entire domain. Figure 4 shows an example time course of $\Delta \left(L^{-1}\right)$. The data shown were generated from the same simulation illustrated in Figure 2, where the bath concentration of sodium was 0.02 M, $K_{\mathrm{Ca}}>K_{\mathrm{Na}}$, and binding sites were sparse.

Figure 4 shows several behaviors. At extremely short times, prior to approximately 0.2 s, $\Delta \left(L^{-1}\right)$ decreases rapidly, indicating a rapid propagation of front location. At longer times, after approximately 1.4 s, we again see a rapid decrease in $\Delta \left(L^{-1}\right)$, corresponding to the front reaching the right end of the domain (this occurs in finite time, causing $\Delta \left(L^{-1}\right)$ to reach zero, which cannot be illustrated on the semilog plot). It is worth noting that the front approaching the right hand end of the domain approximately corresponds to the transition between the “short time” and “long time” behaviors seen in Figure 3 (see dashed blue line). However, at intermediate times, we see an approximately exponential decrease in $\Delta \left(L^{-1}\right)$ (again, indicated by an approximately linear behavior on the semilog plot). To identify the rate of this exponential decrease, we perform a linear fit to the quantity

$$\tilde{L}=\ln\left(\Delta \left(L^{-1}\right)\right).$$

The slope of this linear fit can be interpreted as the exponential decay rate γ (see Equation (23)). To eliminate the contribution of the extremely early time start-up behavior, as well as the effects of the front reaching the right end of the domain, we only use data points between 0.2 s and 0.8 s. For reference, Figure 4 shows a dashed line indicating a purely exponential behavior with the decay rate γ=2.39 s${}^{-1}$, which is the value calculated from the numerical simulation.

**Figure 5:**
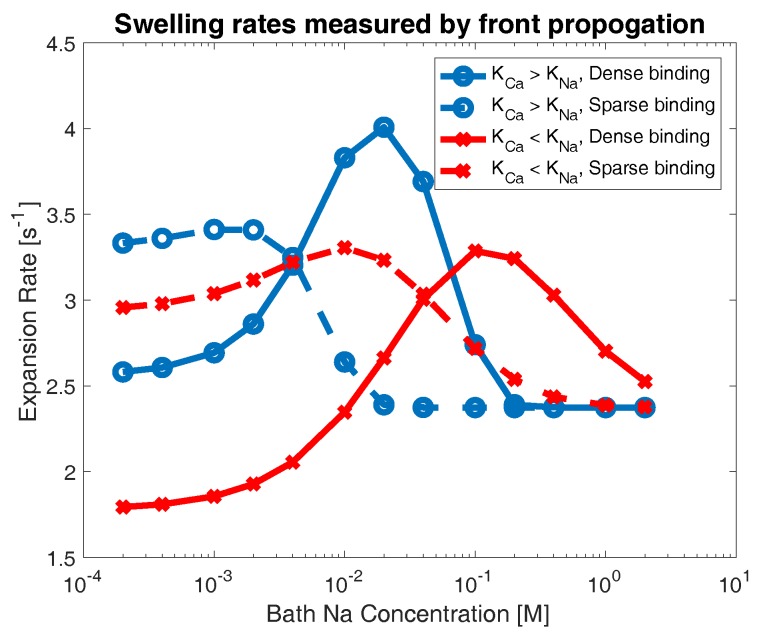
Computed swelling rate γ as the bath concentration of sodium is varied. Data are shown for sparse (dashed) and dense (solid) network binding in the cases where sodium (blue circles) and calcium (red crosses) binding is preferred.

Figure 5 shows the calculated decay rate γ across all experiments. Red curves indicate cases where calcium binding is preferred ($K_{\mathrm{Ca}}<K_{\mathrm{Na}}$), while blue curves indicate networks that chemically prefer sodium binding ($K_{\mathrm{Na}}<K_{\mathrm{Ca}}$). Dashed lines indicate networks with sparse binding sites ($\tilde{z}=0.1$) and solid lines indicate networks with dense binding sites ($\tilde{z}=1$). For extreme concentrations of sodium in the bath, the behavior of gel swelling is relatively easy to characterize. At very high bath concentrations of sodium, all networks appear to swell at the same rate. In this limit, bath sodium is so prevalent that all calcium crosslinks on the network are rapidly replaced with sodium binding (regardless of the relative size of dissociation constants, or number of binding sites), and the networks all swell in an identical manner. In the limit of low sodium concentration, we observe two clear patterns: a network with dense binding sites swells more slowly than one with sparse binding, and a network that chemically prefers calcium bonds swells more slowly than one that prefers sodium bonds. To understand this behavior, note that, if the bath concentration of sodium is very low, then the total number of sodium ions in the domain is severely limited. This means that a network with a high density of binding sites cannot break a large proportion of calcium crosslinks in favor of sodium bonds, and thus will not swell very rapidly (i.e., solid curves indicate slower swelling than their dashed counterparts in Figure 5). By a similar logic, if the number of sodium ions is small, then a network that prefers calcium bonds will not break crosslinks in favor of sodium binding, and will not swell rapidly (i.e., red curves indicate slower swelling than their blue counterparts in Figure 5).

However, at moderate concentrations of sodium, we see regimes where these patterns are reversed. For networks that prefer sodium bonds (blue curves), for sodium concentrations between approximately $4\times 10^{-3}$ and $2\times 10^{-1}$ M, the network with dense binding sites swells more rapidly than the network with sparse binding sites. A similar behavior is observed for networks that prefer calcium bonds (red curves), though at higher bath concentrations of sodium (approximately $4\times 10^{-2}$ to 2 M), as more sodium ions are required to displace calcium bonds. Similarly, for certain regimes of moderate bath sodium concentrations, we see that networks which prefer calcium bonds (red curves) actually swell faster than networks which prefer sodium bonds (blue curves). This behavior occurs between roughly $4\times 10^{-3}$ and $4\times 10^{-1}$ M for sparse binding sites and $2\times 10^{-1}$ and 2 M for dense binding sites. This result is counter-intuitive, as one would suppose that chemically preferring calcium bonds would always retard the breaking of calcium crosslinks, and thus result in slower swelling behavior.

## 4. Discussion

In this paper, we have presented the first investigation of the dynamic swelling behavior of the model of polyelectrolyte gels first derived in [12]. We characterized the early-time swelling rate of the gel network as a function of the chemical parameters that govern the network’s ability and propensity to form crosslinks via calcium bonds, as well as the bath concentration of sodium. Our primary focus was on how the loss of calcium crosslinks alters the energetic landscape of the gel network, thereby driving a swelling event. The fact that swelling rate depends in a non-monotonic way on the concentration of sodium in the bath is a major result of this work. We do not currently have a simple explanation for this counter-intuitive behavior, and future investigations will address this issue. Experimental validation of this prediction would be exciting, but we know of no directly comparable studies. Experimental data would also provide an opportunity to estimate several parameters in the model (specifically, the interaction energies $\epsilon_{k}$) for which we have no reliable estimates.

Finally, we note that, for the sake of simplicity, we chose to treat the rheology of the mucus network as constant and viscous. However, it is known that mucus networks may exhibit a wide range of viscoelastic behaviors depending on the ionic milieu and other factors [21,22,23], including the presence of permanent covalent crosslinking within the gel network [24]. It may be that the formation and breakage of calcium crosslinks alters the rheology of mucus gel in a manner which we do not account for here. If so, the dynamics of swelling are likely governed by a complex interplay between the changing the internal energy and the evolving rheology of the gel mixture as its crosslinking structure varies in space and time. Such considerations are beyond the scope of this work, but may be included in future modeling attempts and potentially fully characterize the ionic regulation of mucus swelling in the human stomach.

## Figures and Tables

**Table 1:** Parameters that do not vary between sets of experiments.

Parameter	Value
$\epsilon_{1}$	−45
$\epsilon_{2}$	25
$\epsilon_{3}$	−0.5
$\epsilon_{4}$	0
*N*	6
inner $\theta_{n}$	0.5
bath $\theta_{n}$	$1\times 10^{-9}$
Total bath Ca	1 mM
Total bath Na	0.2–200 mM
Total bath Cl	0.2–202 mM
$D_{\mathrm{Na}}$	$2.5\times 10^{-5}$ cm${}^{2}$/s
$D_{\mathrm{Ca}}$	$2.5\times 10^{-5}$ cm${}^{2}$/s
$D_{\mathrm{Cl}}$	$2.5\times 10^{-5}$ cm${}^{2}$/s

**Table 2:** Parameters that vary between experiments.

Parameter	$K_{\mathrm{Ca}}>K_{\mathrm{Na}}$,	$K_{\mathrm{Ca}}>K_{\mathrm{Na}}$,	$K_{\mathrm{Ca}}<K_{\mathrm{Na}}$,	$K_{\mathrm{Ca}}<K_{\mathrm{Na}}$,
	Dense Binding	Sparse Binding	Dense Binding	Sparse Binding
Total inner Ca	250 mM	25.3 mM	250 mM	25.3 mM
Total inner Na	5 mM	1 mM	5 mM	1 mM
Total inner Cl	5 mM	1.6 mM	5 mM	1.6 mM
$k_{\mathrm{Ca}}^{\mathrm{on}}$	$1\times 10^{6}$ M${}^{-1}$s${}^{-1}$	$1\times 10^{6}$ M${}^{-1}$s${}^{-1}$	$1\times 10^{7}$ M${}^{-1}$s${}^{-1}$	$1\times 10^{7}$ M${}^{-1}$s${}^{-1}$
$k_{\mathrm{Ca}}^{\mathrm{off}}$	$1\times 10^{3}$ s${}^{-1}$	$1\times 10^{3}$ s${}^{-1}$	$1\times 10^{3}$ s${}^{-1}$	$1\times 10^{3}$ s${}^{-1}$
$k_{\mathrm{Na}}^{\mathrm{on}}$	$5\times 10^{6}$ M${}^{-1}$s${}^{-1}$	$5\times 10^{6}$ M${}^{-1}$s${}^{-1}$	$5\times 10^{5}$ M${}^{-1}$s${}^{-1}$	$5\times 10^{5}$ M${}^{-1}$s${}^{-1}$
$k_{\mathrm{Na}}^{\mathrm{off}}$	$5\times 10^{2}$ s${}^{-1}$	$5\times 10^{2}$ s${}^{-1}$	$5\times 10^{2}$ s${}^{-1}$	$5\times 10^{2}$ s${}^{-1}$
$\tilde{z}$	1 M	0.1 M	1 M	0.1 M

**Figure A1:**
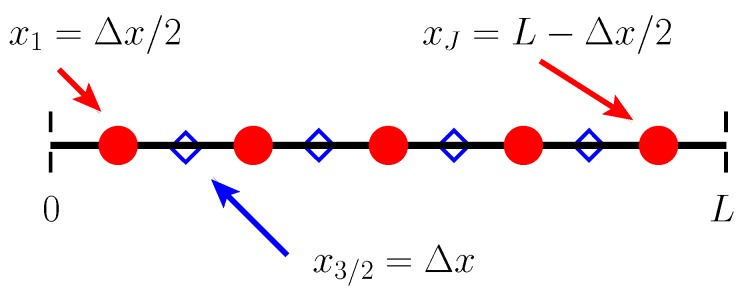
A schematic representation of our computational grid for J=5. Dashed vertical black lines indicate the boundaries of the computational domain. Circles indicate cell centers, while diamonds indicate cell edges.

## Appendix A

### Appendix A.1. Numerical Scheme

Here, we outline the numerical scheme used to simulate our system of of equations. To solve the momentum equations while respecting the volume-averaged incompressibility constraint (Equations (3) to (5)), we utilize a relatively standard second order finite difference method, in conjunction with the “ε-regularization” outlined in [25]. To evolve the volume fractions of network and solvent, we utilize a variation of second-order finite volume method (with van Leer limiter) [26]. To evolve the equations which govern ion transport and chemistry, we use a semi-implicit variation of the electrodiffusive solution scheme described in [27].

We begin by discretizing space using a so-called “staggered grid”. Two collections of spatial points are defined, and various quantities more naturally “live” at each. The first collection of points we refer to as “cell centers,” and they are defined by

(A1) $$x_{j}=\left(j-1/2\right)\;\Delta x\;j=1,2,3\ldots J.$$

The second collection of points are referred to as “cell edges” and are defined by

(A2) $$x_{j}=\left(j-1/2\right)\;\Delta x\;j=\frac{3}{2},\frac{5}{2},\ldots J-1/2,$$

where Δx=L/J is the spatial resolution of the grid. There are in total J−1 cell edges and *J* cell centers. A schematic of the spatial discretization is shown in Figure A1. Finally, we have a single temporal discretization

(A3) $$t_{k}=k\;\Delta t,\;k=0,1,\ldots$$

We approximate ionic concentrations (bound and dissolved), as well as volume fractions and pressure at cell centers. Where necessary, we use a second subscript *j* to denote the spatial location where an approximation takes place, while a superscript *k* denotes the temporal location.

(A4) $$C_{i,\;j}^{k}\approx c_{i}\left(x_{j},t_{k}\right),\;i=\mathrm{Na},\mathrm{Ca},\mathrm{Cl}.$$

(A5) $$B_{i,\;j}^{k}\approx b_{i}\left(x_{j},t_{k}\right),\;i=\mathrm{Na},\mathrm{Ca},C2.$$

(A6) $$\Theta_{i,\;j}^{k}\approx \theta_{i}\left(x_{j},t_{k}\right),\;i=s,n.$$

(A7) $$P_{j}^{k}\approx p\left(x_{j},t_{k}\right).$$

Because concentrations are defined at cell centers, particle fractions are as well.

(A8) $${\hat{\phi }}_{i,\;j}^{k}\approx \phi_{j}\left(x_{j},t_{k}\right),\;i=\mathrm{Na},\mathrm{Ca},\mathrm{Cl}.$$

Conversely, we approximate velocities, potential gradients, and the electric potential gradient at cell edges.

(A9) $$U_{i,\;j}^{k}\approx {\vec{u}}_{i}\left(x_{j},t_{k}\right),\;i=s,n.$$

For brevity, we introduce the quantity $V_{i}$ to represent the potential gradient associated with species *i*, and Φ to represent the electric potential gradient.

(A10) $$V_{i,\;j}^{k}\approx \nabla \mu_{i}\left(x_{j},t_{k}\right),$$

(A11) $$\Phi_{j}^{k}\approx \nabla \Psi \left(x_{j},t_{k}\right).$$

The overall time integration scheme can broadly be described in the following steps carried out at each timestep:

1. Given ionic concentrations ($C_{i,\;j}^{k}$ and $B_{i,\;j}^{k}$), electric potential gradient ($\Phi_{j}^{k}$), and both volume fractions ($\Theta_{i,\;j}^{k}$), evaluate the potential gradients which act on the solvent and network phase ($V_{i,\;j}^{k}$).
2. Given the potential gradients on each phase ($V_{i,\;j}^{k}$), solve the momentum equations to determine the network and solvent velocities ($U_{i,\;j}^{k}$).
3. Given their respective velocity fields, update the solvent and network volume fraction ($\Theta_{i,\;j}^{k+1}$).
4. Given the two transport velocities and volume fractions ($U_{i,\;j}^{k}$ and $\Theta_{i,\;j}^{k+1}$), evolve the ionic concentrations while simultaneously solving for a new electric potential gradient ($C_{i,\;j}^{k+1}$, $B_{i,\;j}^{k+1}$ and $\Phi_{j}^{k+1}$).

#### Appendix A.1.1. Momentum Solve

To solve the momentum equations for the two velocity fields, we use the ε-regularization described in [25]. This method is designed to avoid complications where the linear operators in Equations (4) and (5) become ill defined in regions where $\theta_{n}\approx 0$. Simply put, we define “regularized” volume fractions which avoids such an issue

(A12) $${\hat{\Theta }}_{n\;j}^{k}=\left\{\begin{array}{cc} \Theta_{n,\;j}^{k}+\varepsilon & \mathrm{if}\;\min_{j=1\ldots J}\Theta_{n,\;j}^{k}<\varepsilon \\ \Theta_{n,\;j}^{k} & \mathrm{otherwise}\; \end{array}\right..$$

(A13) $${\hat{\Theta }}_{s\;j}^{k}=1-{\hat{\Theta }}_{n\;j}^{k}.$$

We then use these volume fractions in discrete versions of the momentum and volume-averaged incompressibility constraint to avoid any poorly scaled linear operators. These “regularized” volume fractions are not used later when we update the actual volume fractions, but are simply used to create a regularization of the elliptic operators that define the velocity fields. Using standard second order finite difference schemes and moving all potentials (aside from pressure) to the right hand side, we can write Equations (4) and (5) as a set of linear equations to solve for the unknown velocities. The discrete version of Equation (4) at cell edges takes the form

(A14) $$\begin{array}{c} \frac{\nu_{n}}{\Delta x^{2}}\left({\hat{\Theta }}_{n,\;j+1/2}^{k}U_{n,\;j+1}^{k}-\left({\hat{\Theta }}_{n,\;j+1/2}^{k}+{\hat{\Theta }}_{n,\;j-1/2}^{k}\right)U_{n,\;j}^{k}+{\hat{\Theta }}_{n,\;j-1/2}^{k}U_{n,\;j-1}^{k}\right)\\ -\xi \frac{\left({\hat{\Theta }}_{n,\;j+1/2}^{k}+{\hat{\Theta }}_{n,\;j-1/2}^{k}\right)}{2}\frac{\left({\hat{\Theta }}_{s,\;j+1/2}^{k}+{\hat{\Theta }}_{s,\;j-1/2}^{k}\right)}{2}\left(U_{n,\;j}^{k}-U_{s,\;j}^{k}\right)\\ -\frac{\left({\hat{\Theta }}_{n,\;j+1/2}^{k}+{\hat{\Theta }}_{n,\;j-1/2}^{k}\right)}{2\Delta x}\left(P_{j+1/2}^{k}-P_{j-1/2}^{k}\right)=\frac{\left({\hat{\Theta }}_{n,\;j+1/2}^{k}+{\hat{\Theta }}_{n,\;j-1/2}^{k}\right)}{2}V_{n,\;j}^{k}. \end{array}$$

At cell edges which are adjacent to the domain boundary (j=3/2 and j=J−1/2), these equations contain terms which are not represented on our numerical grid. However, the closed domain and corresponding no-flux conditions on solvent and network imply that $U_{n,\;1/2}^{k}=U_{n,\;J+1/2}^{k}=0$. Similarly, $U_{s,\;1/2}^{k}=U_{s,\;J+1/2}^{k}=0$. For j=3/2…J−1/2,

(A15) $$\begin{array}{c} \frac{\nu_{s}}{\Delta x^{2}}\left({\hat{\Theta }}_{s,\;j+1/2}^{k}U_{s,\;j+1}^{k}-\left({\hat{\Theta }}_{s,\;j+1/2}^{k}+{\hat{\Theta }}_{s,\;j-1/2}^{k}\right)U_{s,\;j}^{k}+{\hat{\Theta }}_{s,\;j-1/2}^{k}U_{s,\;j-1}^{k}\right)\\ -\xi \frac{\left({\hat{\Theta }}_{n,\;j+1/2}^{k}+{\hat{\Theta }}_{n,\;j-1/2}^{k}\right)}{2}\frac{\left({\hat{\Theta }}_{s,\;j+1/2}^{k}+{\hat{\Theta }}_{s,\;j-1/2}^{k}\right)}{2}\left(U_{s,\;j}^{k}-U_{n,\;j}^{k}\right)\\ -\frac{\left({\hat{\Theta }}_{s,\;j+1/2}^{k}+{\hat{\Theta }}_{s,\;j-1/2}^{k}\right)}{2\Delta x}\left(P_{j+1/2}^{k}-P_{j-1/2}^{k}\right)\\ =\frac{\left({\hat{\Theta }}_{s,\;j+1/2}^{k}+{\hat{\Theta }}_{s,\;j-1/2}^{k}\right)}{2}V_{s,\;j}^{k}+\frac{\left({\hat{\Theta }}_{s,\;j+1/2}^{k}+{\hat{\Theta }}_{s,\;j-1/2}^{k}\right)}{2}\sum_{i=\mathrm{Na},\mathrm{Ca},\mathrm{Cl}.}\frac{\left({\hat{\phi }}_{i,\;j+1/2}^{k}+{\hat{\phi }}_{i,\;j-1/2}^{k}\right)}{2}V_{i,\;j}^{k}. \end{array}$$

Again, we stipulate that $U_{s,\;1/2}^{k}=U_{s,\;J+1/2}^{k}=0$. The discrete form of the incompressibility constraint is enforced “at” the cell centers. Therefore, for j=1…J, we have

(A16) $$\begin{array}{c} \frac{1}{\Delta x}\left(\frac{\left({\hat{\Theta }}_{s,\;j+1}^{k}+{\hat{\Theta }}_{s,\;j}^{k}\right)}{2}U_{s,\;j+1/2}^{k}+\frac{\left({\hat{\Theta }}_{n,\;j+1}^{k}+{\hat{\Theta }}_{n,\;j}^{k}\right)}{2}U_{n,\;j+1/2}^{k}\right.\\ \;\left.-\frac{\left({\hat{\Theta }}_{s,\;j}^{k}+{\hat{\Theta }}_{s,\;j-1}^{k}\right)}{2}U_{s,\;j-1/2}^{k}-\frac{\left({\hat{\Theta }}_{n,\;j}^{k}+{\hat{\Theta }}_{n,\;j-2}^{k}\right)}{2}U_{n,\;j-1/2}^{k}\right)=0. \end{array}$$

Equations (A14) to (A16) are a set of 3J−2 linear equations for the unknowns $U_{s,\;j}^{k}$, $U_{n,\;j}^{k}$ (for j=3/2…J−1/2) and $P_{j}^{k}$ (for j=1…J). Solving these equations allows us to determine the velocity fields and pressure at the *k*-th time step.

#### Appendix A.1.2. Volume Fraction Advection

Once the velocities and pressure field have been determined, we use the advection equations (Equations (1) and (2)) to update each volume fraction. To do this, we use a modification of a standard high-order advection scheme. Given solvent volume fractions at the cell centers ($\Theta_{s,\;j}^{k}$, j=1…J) and velocities at cell edges ($U_{s,\;j}^{k}$, j=3/2…J−1/2), we define fluxes at the cell edges using a van Leer limited high order method [26] ($F_{s,\;j}^{k}$, j=3/2…J−1/2). We then update the volume fraction via the following relation:

(A17) $$\Theta_{s,\;j}^{k+1}=\Theta_{s,\;j}^{k}+\frac{\Delta t}{\Delta x}\left(F_{s,\;j-1/2}^{k}-F_{s,\;j+1/2}^{k}\right),\;j=1\ldots J.$$

To enforce the no-flux boundary conditions consistent with a closed domain, we assume that $F_{s,\;1/2}^{k}=F_{s,\;J+1/2}^{k}=0$. Finally, we update the volume fraction of network via

(A18) $$\Theta_{n,\;j}^{k+1}=1-\Theta_{s,\;j}^{k+1}.$$

At this point, the concentration of dissolved ions must be numerically corrected. To illustrate, consider the concentration of chloride in the domain. The evolution equation (Equation (9)) (and the derivation of our model in [12]) conserve the total amount of chloride in the domain

(A19) $$\int_{0}^{L}\theta_{s}c_{\mathrm{Cl}}\;dx=\mathrm{const}.$$

Therefore, our numerical scheme must be conservative as well, preserving the numerical integral

(A20) $$\sum_{j=1\ldots J}\Theta_{s,\;j}^{k}C_{\mathrm{Cl},\;j}^{k}\Delta x=\mathrm{const}.$$

This is particularly important in problems of electrodiffusion, as a non-conservative numerical scheme may lead to a local imbalance of charge within the domain, which will result in extremely large electric potential gradients that may prove numerically untenable. However, even though Equation (A17) is a conservative scheme for the quantity $\Theta_{s}$, the update may have altered the discrete integral in Equation (A20). To correct this, we first calculate the flux quantities ${\tilde{F}}_{i,\;j}^{k}$ (at cell edges) for each ion *i* by using the same van Leer limited high order method on the quantity $\Theta_{s,\;j}^{k}C_{i,\;j}^{k}$ (again, using solvent velocities). We then define the corrected concentration

(A21) $$C_{i\;,j}^{*}=\frac{C_{i,\;j}^{k}\Theta_{s,\;j}^{k}+\frac{\Delta t}{\Delta x}\left({\tilde{F}}_{i,\;j-1/2}^{k}-{\tilde{F}}_{i,\;j+1/2}^{k}\right)}{\Theta_{s,\;j}^{k+1}},\;j=1\ldots J.$$

This corrected concentration ensures the discrete conservation of each ionic species

(A22) $$\sum_{j=1\ldots J}\Theta_{s,\;j}^{k}C_{i,\;j}^{k}\Delta x=\sum_{j=1\ldots J}\Theta_{s,\;j}^{k+1}C_{i,\;j}^{*}\Delta x.$$

At this point, having corrected any ionic imbalances that the volume fraction advection step may have created, we are able to take a time step of the electrodiffusive equations.

#### Appendix A.1.3. Ion Update

To time-step the evolution equations for ionic species, we use a methodology similar to that employed in [27]. The goal is to use an implicit–explicit (IMEX) time integration technique on all ionic species simultaneously, to avoid any further time-splitting errors. Unfortunately, the electric flux term and several reaction terms involve non-linear products of ionic concentrations and/or the electric potential gradient. Therefore, we treat these terms semi-implicitly in time. To do so requires extrapolating “forward” in time. We use first order extrapolation whenever possible, and zeroth order whenever we must (i.e., at the very first time step). Doing so allows us to define the quantities

(A23) $${\tilde{C}}_{i,\;j}^{k+1}=\left\{\begin{array}{cc} 2C_{i,\;j}^{*}-C_{i,\;j}^{k-1}, & n\geq 1\\ C_{i,\;j}^{*}, & n=0 \end{array}\right.,\;i=\mathrm{Na},\mathrm{Ca},\mathrm{Cl},$$

as well as

(A24) $${\tilde{B}}_{i,\;j}^{k+1}=\left\{\begin{array}{cc} 2B_{i,\;j}^{k}-B_{i,\;j}^{k-1}, & n\geq 1\\ B_{i,\;j}^{*}, & n=0 \end{array}\right.,\;i=\mathrm{Na},\mathrm{Ca},C2,$$

We then define $F_{i,\;j}^{k+1}$ as the fluxes of $C_{i,\;j}^{*}$ due to solvent velocity (using the same van Leer limited method) and $G_{i,\;j}^{k+1}$ as the fluxes of $B_{i,\;j}^{k}$ due to network velocity.

We are now able to discretize Equations (7) to (9) for the evolution of each of the three dissolved ionic species. At cell centers which are not adjacent to the boundary (j=2,3,…J−1), we have

(A25) $$\begin{array}{c} \frac{C_{\mathrm{Na},\;j}^{k+1}-C_{\mathrm{Na},\;j}^{k}}{\Delta t}+\frac{1}{\Delta x}\left(F_{\mathrm{Na},\;j+1/2}^{k}-F_{\mathrm{Na},\;j-1/2}^{k}\right)=\\ \frac{D_{\mathrm{Na}}}{\Theta_{s,\;j}^{k+1}\Delta x^{2}}\left[\left(\frac{\Theta_{s,\;j+1}^{k+1}+\Theta_{s,\;j}^{k+1}}{2}\right)C_{\mathrm{Na},\;j+1}^{k+1}-\left(\frac{\Theta_{s,\;j+1}^{k+1}+2\Theta_{s,\;j}^{k+1}+\Theta_{s,\;j-1}^{k+1}}{2}\right)C_{\mathrm{Na},\;j}^{k+1}+\left(\frac{\Theta_{s,\;j}^{k+1}+\Theta_{s,\;j-1}^{k+1}}{2}\right)C_{\mathrm{Na},\;j-1}^{k+1}\right]\\ +\frac{D_{\mathrm{Na}}z_{\mathrm{Na}}}{\Theta_{s,\;j}^{k}\Delta x}\left[\left(\frac{\Theta_{s,\;j+1}^{k+1}+\Theta_{s,\;j}^{k+1}}{2}\right)\left(\frac{{\tilde{C}}_{\mathrm{Na},\;j+1}^{k+1}+{\tilde{C}}_{\mathrm{Na},\;j}^{k+1}}{2}\right)\Phi_{j+1/2}^{k+1}-\left(\frac{\Theta_{s,\;j}^{k+1}+\Theta_{s,\;j-1}^{k+1}}{2}\right)\left(\frac{{\tilde{C}}_{\mathrm{Na},\;j}^{k+1}+{\tilde{C}}_{\mathrm{Na},\;j-1}^{k+1}}{2}\right)\Phi_{j-1/2}^{k+1}\right]\\ -k_{\mathrm{Na}}^{\mathrm{on}}\Theta_{n,\;j}^{k+1}\left(\tilde{z}\Theta_{n,\;j}^{k+1}-B_{\mathrm{Na},\;j}^{k+1}-B_{\mathrm{Ca},\;j}^{k+1}-2B_{C2,\;j}^{k+1}\right){\tilde{C}}_{\mathrm{Na},\;j}^{k+1}+k_{\mathrm{Na}}^{\mathrm{off}}{\left(\Theta_{s,\;j}^{k+1}\right)}^{2}B_{\mathrm{Na},\;j}^{k+1}. \end{array}$$

At the first cell center (j=1), the no flux boundary condition yields

(A26) $$\begin{array}{c} \frac{C_{\mathrm{Na},\;1}^{k+1}-C_{\mathrm{Na},\;1}^{k}}{\Delta t}+\frac{1}{\Delta x}\left(F_{\mathrm{Na},\;3/2}^{k}\right)=\frac{D_{\mathrm{Na}}}{\Theta_{s,\;1}^{k+1}\Delta x^{2}}\left[\left(\frac{\Theta_{s,\;2}^{k+1}+\Theta_{s,\;1}^{k+1}}{2}\right)C_{\mathrm{Na},\;2}^{k+1}-\left(\frac{\Theta_{s,\;2}^{k+1}+\Theta_{s,\;j}^{k+1}}{2}\right)C_{\mathrm{Na},\;1}^{k+1}\right]\\ +\frac{D_{\mathrm{Na}}z_{\mathrm{Na}}}{\Theta_{s,\;1}^{k}\Delta x}\left[\left(\frac{\Theta_{s,\;2}^{k+1}+\Theta_{s,\;1}^{k+1}}{2}\right)\left(\frac{{\tilde{C}}_{\mathrm{Na},\;2}^{k+1}+{\tilde{C}}_{\mathrm{Na},\;1}^{k+1}}{2}\right)\Phi_{3/2}^{k+1}\right]\\ -k_{\mathrm{Na}}^{\mathrm{on}}\Theta_{n,\;1}^{k+1}\left(\tilde{z}\Theta_{n,\;1}^{k+1}-B_{\mathrm{Na},\;1}^{k+1}-B_{\mathrm{Ca},\;1}^{k+1}-2B_{C2,\;1}^{k+1}\right){\tilde{C}}_{\mathrm{Na},\;1}^{k+1}+k_{\mathrm{Na}}^{\mathrm{off}}{\left(\Theta_{s,\;1}^{k+1}\right)}^{2}B_{\mathrm{Na},\;1}^{k+1}. \end{array}$$

At the last cell center (j=J), the no flux boundary condition yields

(A27) $$\begin{array}{c} \frac{C_{\mathrm{Na},\;J}^{k+1}-C_{\mathrm{Na},\;J}^{k}}{\Delta t}+\frac{1}{\Delta x}\left(-F_{\mathrm{Na},\;J-1/2}^{k}\right)=\\ \frac{D_{\mathrm{Na}}}{\Theta_{s,\;J}^{k+1}\Delta x^{2}}\left[-\left(\frac{\Theta_{s,\;J}^{k+1}+\Theta_{s,\;J-1}^{k+1}}{2}\right)C_{\mathrm{Na},\;J}^{k+1}+\left(\frac{\Theta_{s,\;J}^{k+1}+\Theta_{s,\;J-1}^{k+1}}{2}\right)C_{\mathrm{Na},\;J-1}^{k+1}\right]\\ +\frac{D_{\mathrm{Na}}z_{\mathrm{Na}}}{\Theta_{s,\;J}^{k}\Delta x}\left[-\left(\frac{\Theta_{s,\;J}^{k+1}+\Theta_{s,\;J-1}^{k+1}}{2}\right)\left(\frac{{\tilde{C}}_{\mathrm{Na},\;J}^{k+1}+{\tilde{C}}_{\mathrm{Na},\;J-1}^{k+1}}{2}\right)\Phi_{J-1/2}^{k+1}\right]\\ -k_{\mathrm{Na}}^{\mathrm{on}}\Theta_{n,\;J}^{k+1}\left(\tilde{z}\Theta_{n,\;J}^{k+1}-B_{\mathrm{Na},\;J}^{k+1}-B_{\mathrm{Ca},\;J}^{k+1}-2B_{C2,\;J}^{k+1}\right){\tilde{C}}_{\mathrm{Na},\;J}^{k+1}+k_{\mathrm{Na}}^{\mathrm{off}}{\left(\Theta_{s,\;J}^{k+1}\right)}^{2}B_{\mathrm{Na},\;J}^{k+1}. \end{array}$$

Similarly, the equations for the dissolved calcium are (for j=2…J−1)

(A28) $$\begin{array}{c} \frac{C_{\mathrm{Ca},\;j}^{k+1}-C_{\mathrm{Ca},\;j}^{k}}{\Delta t}+\frac{1}{\Delta x}\left(F_{\mathrm{Ca},\;j+1/2}^{k}-F_{\mathrm{Ca},\;j-1/2}^{k}\right)=\\ \frac{D_{\mathrm{Ca}}}{\Theta_{s,\;j}^{k+1}\Delta x^{2}}\left[\left(\frac{\Theta_{s,\;j+1}^{k+1}+\Theta_{s,\;j}^{k+1}}{2}\right)C_{\mathrm{Ca},\;j+1}^{k+1}-\left(\frac{\Theta_{s,\;j+1}^{k+1}+2\Theta_{s,\;j}^{k+1}+\Theta_{s,\;j-1}^{k+1}}{2}\right)C_{\mathrm{Ca},\;j}^{k+1}+\left(\frac{\Theta_{s,\;j}^{k+1}+\Theta_{s,\;j-1}^{k+1}}{2}\right)C_{\mathrm{Ca},\;j-1}^{k+1}\right]\\ +\frac{D_{\mathrm{Ca}}z_{\mathrm{Ca}}}{\Theta_{s,\;j}^{k}\Delta x}\left[\left(\frac{\Theta_{s,\;j+1}^{k+1}+\Theta_{s,\;j}^{k+1}}{2}\right)\left(\frac{{\tilde{C}}_{\mathrm{Ca},\;j+1}^{k+1}+{\tilde{C}}_{\mathrm{Ca},\;j}^{k+1}}{2}\right)\Phi_{j+1/2}^{k+1}-\left(\frac{\Theta_{s,\;j}^{k+1}+\Theta_{s,\;j-1}^{k+1}}{2}\right)\left(\frac{{\tilde{C}}_{\mathrm{Ca},\;j}^{k+1}+{\tilde{C}}_{\mathrm{Ca},\;j-1}^{k+1}}{2}\right)\Phi_{j-1/2}^{k+1}\right]\\ -k_{\mathrm{Ca}}^{\mathrm{on}}\Theta_{n,\;j}^{k+1}\left(\tilde{z}\Theta_{n,\;j}^{k+1}-B_{\mathrm{Na},\;j}^{k+1}-B_{\mathrm{Ca},\;j}^{k+1}-2B_{C2,\;j}^{k+1}\right){\tilde{C}}_{\mathrm{Ca},\;j}^{k+1}+k_{\mathrm{Ca}}^{\mathrm{off}}{\left(\Theta_{s,\;j}^{k+1}\right)}^{2}B_{\mathrm{Ca},\;j}^{k+1}. \end{array}$$

At the first cell center (j=1), the no flux boundary condition yields

(A29) $$\begin{array}{c} \frac{C_{\mathrm{Ca},\;1}^{k+1}-C_{\mathrm{Ca},\;1}^{k}}{\Delta t}+\frac{1}{\Delta x}\left(F_{\mathrm{Ca},\;3/2}^{k}\right)=\frac{D_{\mathrm{Ca}}}{\Theta_{s,\;1}^{k+1}\Delta x^{2}}\left[\left(\frac{\Theta_{s,\;2}^{k+1}+\Theta_{s,\;1}^{k+1}}{2}\right)C_{\mathrm{Ca},\;2}^{k+1}-\left(\frac{\Theta_{s,\;2}^{k+1}+\Theta_{s,\;j}^{k+1}}{2}\right)C_{\mathrm{Ca},\;1}^{k+1}\right]\\ +\frac{D_{\mathrm{Ca}}z_{\mathrm{Ca}}}{\Theta_{s,\;1}^{k}\Delta x}\left[\left(\frac{\Theta_{s,\;2}^{k+1}+\Theta_{s,\;1}^{k+1}}{2}\right)\left(\frac{{\tilde{C}}_{\mathrm{Ca},\;2}^{k+1}+{\tilde{C}}_{\mathrm{Ca},\;1}^{k+1}}{2}\right)\Phi_{3/2}^{k+1}\right]\\ -k_{\mathrm{Ca}}^{\mathrm{on}}\Theta_{n,\;1}^{k+1}\left(\tilde{z}\Theta_{n,\;1}^{k+1}-B_{\mathrm{Na},\;1}^{k+1}-B_{\mathrm{Ca},\;1}^{k+1}-2B_{C2,\;1}^{k+1}\right){\tilde{C}}_{\mathrm{Ca},\;1}^{k+1}+k_{\mathrm{Ca}}^{\mathrm{off}}{\left(\Theta_{s,\;1}^{k+1}\right)}^{2}B_{\mathrm{Ca},\;1}^{k+1}. \end{array}$$

At the last cell center (j=J), the no flux boundary condition yields

(A30) $$\begin{array}{c} \frac{C_{\mathrm{Ca},\;J}^{k+1}-C_{\mathrm{Ca},\;J}^{k}}{\Delta t}+\frac{1}{\Delta x}\left(-F_{\mathrm{Ca},\;J-1/2}^{k}\right)=\\ \frac{D_{\mathrm{Ca}}}{\Theta_{s,\;J}^{k+1}\Delta x^{2}}\left[-\left(\frac{\Theta_{s,\;J}^{k+1}+\Theta_{s,\;J-1}^{k+1}}{2}\right)C_{\mathrm{Ca},\;J}^{k+1}+\left(\frac{\Theta_{s,\;J}^{k+1}+\Theta_{s,\;J-1}^{k+1}}{2}\right)C_{\mathrm{Ca},\;J-1}^{k+1}\right]\\ +\frac{D_{\mathrm{Ca}}z_{\mathrm{Ca}}}{\Theta_{s,\;J}^{k}\Delta x}\left[-\left(\frac{\Theta_{s,\;J}^{k+1}+\Theta_{s,\;J-1}^{k+1}}{2}\right)\left(\frac{{\tilde{C}}_{\mathrm{Ca},\;J}^{k+1}+{\tilde{C}}_{\mathrm{Ca},\;J-1}^{k+1}}{2}\right)\Phi_{J-1/2}^{k+1}\right]\\ -k_{\mathrm{Ca}}^{\mathrm{on}}\Theta_{n,\;J}^{k+1}\left(\tilde{z}\Theta_{n,\;J}^{k+1}-B_{\mathrm{Na},\;J}^{k+1}-B_{\mathrm{Ca},\;J}^{k+1}-2B_{C2,\;J}^{k+1}\right){\tilde{C}}_{\mathrm{Ca},\;J}^{k+1}+k_{\mathrm{Ca}}^{\mathrm{off}}{\left(\Theta_{s,\;J}^{k+1}\right)}^{2}B_{\mathrm{Ca},\;J}^{k+1}. \end{array}$$

Because chloride is not allowed to bind to the network, the equations are relatively simple, as there are no reaction terms.

(A31) $$\begin{array}{c} \frac{C_{\mathrm{Cl},\;j}^{k+1}-C_{\mathrm{Cl},\;j}^{k}}{\Delta t}+\frac{1}{\Delta x}\left(F_{\mathrm{Cl},\;j+1/2}^{k}-F_{\mathrm{Cl},\;j-1/2}^{k}\right)=\\ \frac{D_{\mathrm{Cl}}}{\Theta_{s,\;j}^{k+1}\Delta x^{2}}\left[\left(\frac{\Theta_{s,\;j+1}^{k+1}+\Theta_{s,\;j}^{k+1}}{2}\right)C_{\mathrm{Cl},\;j+1}^{k+1}-\left(\frac{\Theta_{s,\;j+1}^{k+1}+2\Theta_{s,\;j}^{k+1}+\Theta_{s,\;j-1}^{k+1}}{2}\right)C_{\mathrm{Cl},\;j}^{k+1}+\left(\frac{\Theta_{s,\;j}^{k+1}+\Theta_{s,\;j-1}^{k+1}}{2}\right)C_{\mathrm{Cl},\;j-1}^{k+1}\right]\\ +\frac{D_{\mathrm{Cl}}z_{\mathrm{Cl}}}{\Theta_{s,\;j}^{k}\Delta x}\left[\left(\frac{\Theta_{s,\;j+1}^{k+1}+\Theta_{s,\;j}^{k+1}}{2}\right)\left(\frac{{\tilde{C}}_{\mathrm{Cl},\;j+1}^{k+1}+{\tilde{C}}_{\mathrm{Cl},\;j}^{k+1}}{2}\right)\Phi_{j+1/2}^{k+1}-\left(\frac{\Theta_{s,\;j}^{k+1}+\Theta_{s,\;j-1}^{k+1}}{2}\right)\left(\frac{{\tilde{C}}_{\mathrm{Cl},\;j}^{k+1}+{\tilde{C}}_{\mathrm{Cl},\;j-1}^{k+1}}{2}\right)\Phi_{j-1/2}^{k+1}\right]. \end{array}$$

At the first cell center (j=1), the no flux boundary condition yields

(A32) $$\begin{array}{cc} \frac{C_{\mathrm{Cl},\;1}^{k+1}-C_{\mathrm{Cl},\;1}^{k}}{\Delta t}+\frac{1}{\Delta x}\left(F_{\mathrm{Cl},\;3/2}^{k}\right)= & \frac{D_{\mathrm{Cl}}}{\Theta_{s,\;1}^{k+1}\Delta x^{2}}\left[\left(\frac{\Theta_{s,\;2}^{k+1}+\Theta_{s,\;1}^{k+1}}{2}\right)C_{\mathrm{Cl},\;2}^{k+1}-\left(\frac{\Theta_{s,\;2}^{k+1}+\Theta_{s,\;j}^{k+1}}{2}\right)C_{\mathrm{Cl},\;1}^{k+1}\right]\\ & +\frac{D_{\mathrm{Cl}}z_{\mathrm{Cl}}}{\Theta_{s,\;1}^{k}\Delta x}\left[\left(\frac{\Theta_{s,\;2}^{k+1}+\Theta_{s,\;1}^{k+1}}{2}\right)\left(\frac{{\tilde{C}}_{\mathrm{Cl},\;2}^{k+1}+{\tilde{C}}_{\mathrm{Cl},\;1}^{k+1}}{2}\right)\Phi_{3/2}^{k+1}\right]. \end{array}$$

At the last cell center (j=J), the no flux boundary condition yields

(A33) $$\begin{array}{c} \frac{C_{\mathrm{Cl},\;J}^{k+1}-C_{\mathrm{Cl},\;J}^{k}}{\Delta t}+\frac{1}{\Delta x}\left(-F_{\mathrm{Cl},\;J-1/2}^{k}\right)=\\ \frac{D_{\mathrm{Cl}}}{\Theta_{s,\;J}^{k+1}\Delta x^{2}}\left[-\left(\frac{\Theta_{s,\;J}^{k+1}+\Theta_{s,\;J-1}^{k+1}}{2}\right)C_{\mathrm{Cl},\;J}^{k+1}+\left(\frac{\Theta_{s,\;J}^{k+1}+\Theta_{s,\;J-1}^{k+1}}{2}\right)C_{\mathrm{Cl},\;J-1}^{k+1}\right]\\ +\frac{D_{\mathrm{Cl}}z_{\mathrm{Cl}}}{\Theta_{s,\;J}^{k}\Delta x}\left[-\left(\frac{\Theta_{s,\;J}^{k+1}+\Theta_{s,\;J-1}^{k+1}}{2}\right)\left(\frac{{\tilde{C}}_{\mathrm{Cl},\;J}^{k+1}+{\tilde{C}}_{\mathrm{Cl},\;J-1}^{k+1}}{2}\right)\Phi_{J-1/2}^{k+1}\right]. \end{array}$$

Because the equations which govern bound ionic species are not diffusive, they are quite a bit simpler. For sodium, we have (j=1…J)

(A34) $$\begin{array}{c} \frac{B_{\mathrm{Na},\;j}^{k+1}-B_{\mathrm{Na},\;j}^{k}}{\Delta t}+\frac{1}{\Delta x}\left(G_{\mathrm{Na},\;j+1/2}^{k}-G_{\mathrm{Na},\;j-1/2}^{k}\right)=\\ +k_{\mathrm{Na}}^{\mathrm{on}}\Theta_{n,\;j}^{k+1}\left(\tilde{z}\Theta_{n,\;j}^{k+1}-B_{\mathrm{Na},\;j}^{k+1}-B_{\mathrm{Ca},\;j}^{k+1}-2B_{C2,\;j}^{k+1}\right){\tilde{C}}_{\mathrm{Na},\;j}^{k+1}-k_{\mathrm{Na}}^{\mathrm{off}}{\left(\Theta_{s,\;j}^{k+1}\right)}^{2}B_{\mathrm{Na},\;j}^{k+1}. \end{array}$$

The equations for singly- and doubly-bound calcium, respectively, are

(A35) $$\begin{array}{c} \frac{B_{\mathrm{Ca},\;j}^{k+1}-B_{\mathrm{Ca},\;j}^{k}}{\Delta t}+\frac{1}{\Delta x}\left(G_{\mathrm{Ca},\;j+1/2}^{k}-G_{\mathrm{Ca},\;j-1/2}^{k}\right)=\\ +k_{\mathrm{Ca}}^{\mathrm{on}}\Theta_{n,\;j}^{k+1}\left(\tilde{z}\Theta_{n,\;j}^{k+1}-B_{\mathrm{Na},\;j}^{k+1}-B_{\mathrm{Ca},\;j}^{k+1}-2B_{C2,\;j}^{k+1}\right){\tilde{C}}_{\mathrm{Ca},\;j}^{k+1}-k_{\mathrm{Ca}}^{\mathrm{off}}{\left(\Theta_{s,\;j}^{k+1}\right)}^{2}B_{\mathrm{Ca},\;j}^{k+1}\\ -\frac{1}{2}k_{\mathrm{Ca}}^{\mathrm{on}}\left(\tilde{z}\Theta_{n,\;j}^{k+1}-B_{\mathrm{Na},\;j}^{k+1}-B_{\mathrm{Ca},\;j}^{k+1}-2B_{C2,\;j}^{k+1}\right){\tilde{B}}_{\mathrm{Ca},\;j}^{k+1}+2k_{\mathrm{Ca}}^{\mathrm{off}}B_{C2,\;j}^{k+1}, \end{array}$$

(A36) $$\begin{array}{c} \frac{B_{C2,\;j}^{k+1}-B_{C2,\;j}^{k}}{\Delta t}+\frac{1}{\Delta x}\left(G_{C2,\;j+1/2}^{k}-G_{C2,\;j-1/2}^{k}\right)=\\ -\frac{1}{2}k_{\mathrm{Ca}}^{\mathrm{on}}\left(\tilde{z}\Theta_{n,\;j}^{k+1}-B_{\mathrm{Na},\;j}^{k+1}-B_{\mathrm{Ca},\;j}^{k+1}-2B_{C2,\;j}^{k+1}\right){\tilde{B}}_{\mathrm{Na},\;j}^{k+1}+2k_{\mathrm{Ca}}^{\mathrm{off}}B_{C2,\;j}^{k+1}. \end{array}$$

The convention that $G_{i,\;1/2}^{k+1}=G_{i,\;J+1/2}^{k+1}=0$ accounts for the no flux boundary conditions (as all fluxes are advective).

Finally, we have a set of discrete equations which enforce the electroneutrality constraint at cell centers (j=1,2,…J−1).

(A37) $$z_{\mathrm{Na}}\Theta_{s,\;j}^{k+1}C_{\mathrm{Na},\;j}^{k+1}+z_{\mathrm{ca}}\Theta_{s,\;j}^{k+1}C_{\mathrm{Ca},\;j}^{k+1}+z_{\mathrm{Cl}}\Theta_{s,\;j}^{k+1}C_{\mathrm{Cl},\;j}^{k+1}+B_{\mathrm{Na},\;j}^{k+1}+2B_{\mathrm{Ca},\;j}^{k+1}+2B_{C2,\;j}^{k+1}=-\tilde{z}\Theta_{n\;j}^{k+1}.$$

Equations (A25) to (A37) constitute 7J−1 linear equations for the 7J−1 unknown electrodiffusive quantities (*J* each for the $C_{i,\;j}^{k+1}$s and $B_{i,\;j}^{k+1}$s, and J−1 for the $\Phi_{j}^{k+1}$s). Therefore, solving these equations simultaneously evolves the electrodiffusive model one time step.

Notice that we do not enforce electroneutrality at the right most cell center (j=J). This is because the quantities $\Phi_{j}^{k+1}$ may be regarded as Lagrange multipliers on Equations (A25) to (A36) which serve to enforce the constraint in Equation (A37). As we only have J−1 Lagrange multipliers, we are only able to enforce electroneutrality at J−1 cells. However, as our numerical scheme is conservative, the total amount of each ion within the domain is conserved. Therefore, enforcing electroneutrality at all but one cell is guaranteed to enforce electroneutrality at the remaining cell.
